# The lncRNA DANCR promotes breast cancer brain metastasis by acting as a ceRNA for miR-758-3p to regulate PTGS2 expression: DANCR/miR-758-3p/PTGS2 axis drives breast cancer brain metastasis

**DOI:** 10.3724/abbs.2025082

**Published:** 2025-05-15

**Authors:** Sen Li, Yuechao Yang, Zhisu Wang, Liangdong Li, Yang Gao, Yiqun Cao

**Affiliations:** 1 Department of Neurosurgery Fudan University Shanghai Cancer Center Shanghai 200032 China; 2 Department of Oncology Shanghai Medical College Fudan University Shanghai 200032 China

**Keywords:** DANCR, breast cancer, brain metastasis, PTGS2

## Abstract

Brain metastases in breast cancer patients are correlated with markedly lower survival rates than extracranial metastases, highlighting the critical necessity for identifying novel therapeutic targets. The functional involvement of differentiation antagonizing nonprotein coding RNA (DANCR) in the pathogenesis of breast cancer brain metastases (BCBMs) has yet to be fully elucidated. Bioinformatics analyses identify DANCR as a potential specific prognostic biomarker of BCBM. CCK-8, transwell, and wound healing assays are performed to examine the effects of DANCR on the proliferation, migration, and invasion of tumors, along with *in vivo* assays. Mechanistic insights are obtained through quantitative real-time polymerase chain reaction (qRT-PCR), western blot analysis, and dual-luciferase reporter assays. DANCR is markedly upregulated in BCBM and specifically correlates with the prognostic risk of BCBM. DANCR overexpression significantly enhances breast cancer cell proliferation, migration, and invasion. According to low-throughput screening, only the expression of prostaglandin-endoperoxide synthase 2 (PTGS2) consistently varies in parallel with that of DANCR, and PTGS2 silencing reverses DANCR-induced protumor effects *in vitro*. Additionally, in brain metastatic lesions, PTGS2 expression is also elevated in patients with increased DANCR expression. Mechanistically, DANCR and PTGS2 possess a conserved miR-758-3p response element. DANCR directly binds to and sequesters miR-758-3p, thereby alleviating the suppressive effects of miR-758-3p on both DANCR and PTGS2. When the miR-758-3p binding site on DANCR is mutated, this interaction is completely abolished. DANCR drives BCBM by functioning as a miR-758-3p sponge to upregulate PTGS2. Targeting the DANCR/miR-758-3p/PTGS2 axis represents a promising therapeutic approach.

## Introduction

Breast cancer is the most common malignancy among women worldwide. Compared with other types of extracranial metastases, brain metastasis, one of the most severe complications of breast cancer, is associated with a significantly poorer prognosis [1]. With advances in systemic therapies and prolonged survival of breast cancer patients, the incidence of brain metastases has also increased [ 2, 3] . Current estimates indicate that approximately 15% to 30% of breast cancer patients will develop brain metastases [4]. The introduction of anti-erb-b2 receptor tyrosine kinase 2 (HER2) therapies has significantly improved outcomes for patients with HER2-positive breast cancer and brain metastases, extending the median overall survival to 34.4 months [ 5– 7] . However, treatment options for triple-negative BCBM remain limited, with ongoing research focusing on combinations of chemotherapy, immune checkpoint inhibitors, and novel targeted therapies [ 8– 12] .

Through analyses of brain metastasis samples and preclinical models, researchers have identified a number of potential therapeutic targets implicated in BCBM. Elevated expression of vascular endothelial growth factor A (VEGFA), epidermal growth factor receptor (EGFR), and Rous sarcoma oncogene (SRC) enhances the metastatic potential of breast cancer cells to the brain [ 13– 19] . In addition, multiple proteins, including ST6 N-acetylgalactosaminide alpha-2,6-sialyltransferase 5 (ST6GALNAC5), PTGS2, heparin-binding EGF-like growth factor (HBEGF), cathepsin S (CTSS), angiopoietin-2 (ANGPT2), cell migration-inducing hyaluronidase 1 (CEMIP), matrix metallopeptidase 1 (MMP1), cell migration-inducing hyaluronidase 2 (MMP2), sphingosine-1-phosphate receptor 3 (S1P3), and syndecan 1 (SDC1), have been shown to facilitate the transmigration of breast cancer cells across the blood-brain barrier (BBB) [ 19– 27] . Furthermore, protocadherin 7 (PCDH7) and YTH N6-methyladenosine RNA binding protein F3 (YTHDF3) play crucial roles in mediating interactions between metastatic breast cancer cells and astrocytes within the brain microenvironment [ 18, 28] . Despite these advances, effective treatment of BCBM remains a major clinical challenge, and the identification of additional therapeutic targets is urgently needed.

Recent studies have highlighted the significant regulatory roles of long non-coding RNAs (lncRNAs) in tumor progression and metastasis through diverse mechanisms. For example, lncRNA associated with BCBM (Lnc-BM) promotes BCBM by enhancing communication between breast cancer cells and the brain microenvironment [29]. Our previous work demonstrated that lncRNA-cardiac conduction regulatory RNA (CCRR) promotes the formation of gap junctions between metastatic breast cancer cells and astrocytes by upregulating gap junction protein alpha 1 (CX43) expression [30]. Moreover, the exosome-derived lncRNA GS1-600G8.5 has been shown to facilitate BCBM by compromising the integrity of the BBB [31].

In this study, through integrative transcriptomic analysis of multiple independent datasets, we systematically identified differentially expressed lncRNAs associated with brain metastasis-specific signatures in breast cancer, with DANCR emerging as a critical regulatory molecule in this pathological process. Our functional experiments further demonstrated that DANCR plays a critical role in promoting the development and progression of BCBM, highlighting its potential as a novel therapeutic target.

## Materials and Methods

### Bioinformatics analysis

Gene expression data from the GSE14017, GSE18549, GSE83132, GSE180098, GSE223351, GSE12276, and GSE99394 datasets were downloaded from the GEO database ( https://www.ncbi.nlm.nih.gov/geo/). Student’s *t* test and the limma package were used for the statistical analyses [32].

### Tissue specimens

This study adhered to the International Ethical Guidelines for Health-related Research Involving Humans and was approved by the Ethics Committee of Fudan University Shanghai Cancer Center (FUSCC; Shanghai, China; approval No. 050432-4-2307E). Tissue samples from three patients with breast cancer and brain metastases were collected from December 2010 to September 2020 at the FUSCC. A total of 3 primary tumor samples and matched brain metastasis tissues were stored in liquid nitrogen.

### Cell culture and plasmid transfection

The human breast cancer cell lines MCF-7, MDA-MB-231, and MDA-MB-453, as well as the murine breast cancer cell line 4T1, were obtained from the Cell Bank of the Chinese Academy of Sciences (Shanghai, China). The nontumorigenic human breast epithelial cell line MCF-10A and the murine breast cancer cell lines 4T07, 4T1.2, and 66CL4 were generously provided by Dr Huailiang Wu (Fudan University, Shanghai, China). All the cells were cultured in Dulbecco’s modified Eagle’s medium (DMEM; Meilun Biotechnology, Dalian, China) supplemented with 10% fetal bovine serum (FBS; Gibco, Waltham, USA) and 1% penicillin-streptomycin (Invitrogen, Carlsbad, USA) at 37°C in a humidified atmosphere containing 5% CO _2_.

Negative controls (NCs), mimics, and inhibitors of hsa-miR-758-3p and mmu-miR-758-3p, as well as overexpression plasmids for human and murine DANCR (wild-type and mutant) and shRNA plasmids targeting DANCR and PTGS2, along with their respective negative controls (vector and shNC), were synthesized by Tsingke Biological Technology (Shanghai, China). The sequences for all the shRNAs are provided in Supplementary Table S1. Plasmid transfection into MDA-MB-231 and 4T1 cells was performed using Lipofectamine 3000 (Thermo Fisher Scientific, Waltham, USA) according to the manufacturer’s instructions. For stable cell line generation, lentivirus was prepared and applied following the manufacturer’s guidelines, and stable clones were selected using puromycin (1 μg/mL; Thermo Fisher Scientific).

### qRT-PCR analysis

Total RNA was extracted using the Total RNA Extraction kit (TIANGEN, Beijing, China), and reverse transcription was performed with Hifair® II 1st Strand cDNA Synthesis SuperMix (YEASEN, Shanghai, China). By using the Bio-Rad CFX96 real-time PCR detection system (Bio-Rad, Hercules, USA) in a 10 μL reaction volume containing Hieff® qPCR SYBR Green Master Mix (YEASEN), qRT-PCR was conducted. The thermal cycling protocol consisted of initial denaturation at 95°C for 2 min, followed by 39 cycles of 95°C for 10 s and 60°C for 34 s. Primer sequences are listed in Supplementary Table S1.

### CCK-8 assay

The cells were seeded at a density of 1000 cells per well in 96-well plates (Corning, New York, USA) containing 100 μL of complete medium per well and incubated at 37°C in an atmosphere of 5% CO _2_ for 24 h, with 3 replicate wells per group. Cell proliferation was assessed daily for 4 days via a CCK-8 assay kit (Beyotime, Shanghai, China) following the manufacturer’s protocol. After medium was removed from wells, 100 μL of CCK-8 reagent (1:10 diluted with complete medium) was added to each well, followed by incubation at 37°C for 2 h. The absorbance was subsequently measured at 450 nm using a BioTek Synergy H1 microplate reader (Biotek, Winooski, USA).

### Wound healing assay

The cells (6 × 10 ^5^) were seeded in 6-well plates (Corning) for 24 h. A 200 μL pipette tip was used to create a scratch wound across the cell monolayer. The wells were washed with phosphate-buffered saline (PBS; Servicebio, Wuhan, China), and the cells were incubated in medium without FBS. Images were captured immediately after scratching and again after 24 h. The extent of wound closure was quantified using ImageJ software.

### Transwell assay

Matrigel-coated Transwell chambers (Corning) were placed in 24-well plates. A 100 μL cell suspension (2 × 10 ^4^ cells in medium without FBS) was added to the upper chamber, and the lower chamber was filled with complete medium as a chemoattractant. After 24 h of incubation at 37°C, the cells that migrated through the membrane were fixed with 4% paraformaldehyde for 30 min, stained with 1% crystal violet for 15 min, washed, and counted under a Leica DM IL LED inverted microscope (Leica, Wetzlar, Germany).

### Animal experiments

All animal experiments were conducted in accordance with protocols approved by the Animal Experimental Ethical Committee of Fudan University (approval No. 20200306-071). Female BALB/c mice (6 weeks old) were purchased from GemPharmatech Co., Ltd. (Nanjing, China). To establish intracardiac xenograft models, 4T1-Vector and 4T1-Dancr cells (5 × 10 ^4^ cells per mouse) were injected into the left cardiac ventricle. Mice were anesthetized with 1% Pentobarbital in PBS (0.1 mL/10 g), positioned in a supine posture, shaved, and disinfected. A 1-mL syringe was prepared by introducing a 200-μL air gap, followed by filling it with 200 μL cell suspension; any air bubbles were subsequently removed. The syringe was inserted vertically to a depth of 3 mm at the maximum point of impulse. Successful entry into the left ventricle was confirmed by observing bright red blood flow, after which 100 μL of the cell suspension was injected over a period of 10 s. The needle was then slowly withdrawn, and hemostasis was achieved by applying gentle pressure with alcohol cotton balls. Mice were allowed to recover on a warming pad and were subsequently housed under specific pathogen-free conditions. After 12 days, the mice were euthanized after bioluminescence imaging, and the brains were collected for pathological analysis.

### Hematoxylin and eosin (H&E) staining

Harvested brains were fixed in 4% paraformaldehyde for at least 30 min, followed by standard paraffin embedding and sectioning (5 μm thickness). The sections were sequentially deparaffinized by immersing in xylene twice for 10 min each. Subsequently, the sections underwent hydration through graded ethanol solutions: absolute ethanol (5 min), 95% ethanol (5 min), 85% ethanol (5 min), and 70% ethanol (5 min). Following hematoxylin and eosin staining, the sections were re-dehydrated by immersing in 80% ethanol (5 s), 95% ethanol (2 min), and absolute ethanol (2 min). The sections were then cleared in xylene twice for 4 min each. Finally, the slices were air-dried and mounted using neutral gum. The stained sections were examined under a light microscope.

### Western blot analysis

The cells were washed with PBS and lysed on ice in RIPA lysis buffer (Meilun Biotechnology) supplemented with protease inhibitors (Meilun Biotechnology). Equal amounts of protein lysates were separated by SDS-PAGE and transferred onto PVDF membranes (Millipore, Burlington, USA). After blocking with blocking buffer for 1 h at room temperature, the membranes were incubated overnight at 4°C with primary antibodies against GAPDH (ABclonal, Wuhan, China; 1:1000), PTGS2 (ABclonal; 1:1000), PCNA (ABclonal; 1:1000), E-cadherin (ABclonal; 1:1000), N-cadherin (ABclonal; 1:1000) or β-tubulin (ABclonal; 1:1000). The membranes were then washed and incubated with HRP-conjugated secondary antibodies (ABclonal; 1:5000) for 2 h at room temperature. The protein bands were visualized using enhanced chemiluminescence (ECL) detection (Beyotime).

### Immunofluorescence

The tissue sections were deparaffinized and permeabilized before being incubated overnight at 4°C with an anti-PTGS2 antibody (ABclonal; 1:400). After being washed with PBS, the sections were incubated in the dark for 2 h at room temperature with an Alexa Fluor 488-conjugated secondary antibody (Invitrogen; 1:400). Nuclei were counterstained with DAPI-containing mounting medium (Meilun Biotechnology), and the sections were visualized via an X-LIGHT V2 spinning disk confocal system (89 North) attached to a Leica DMi8 microscope (Leica).

### Statistical analysis

All the statistical analyses were conducted via R software (version 4.3.3) and GraphPad Prism version 8.0 (GraphPad Software, La Jolla, USA). For comparisons between two groups, Student’s *t* test was employed, whereas one-way ANOVA was used to assess significant differences among three or more independent groups. Statistical significance was defined as a *P* value less than 0.05.

## Results

### DANCR is associated with brain-specific metastasis in breast cancer

**Figure 1:**
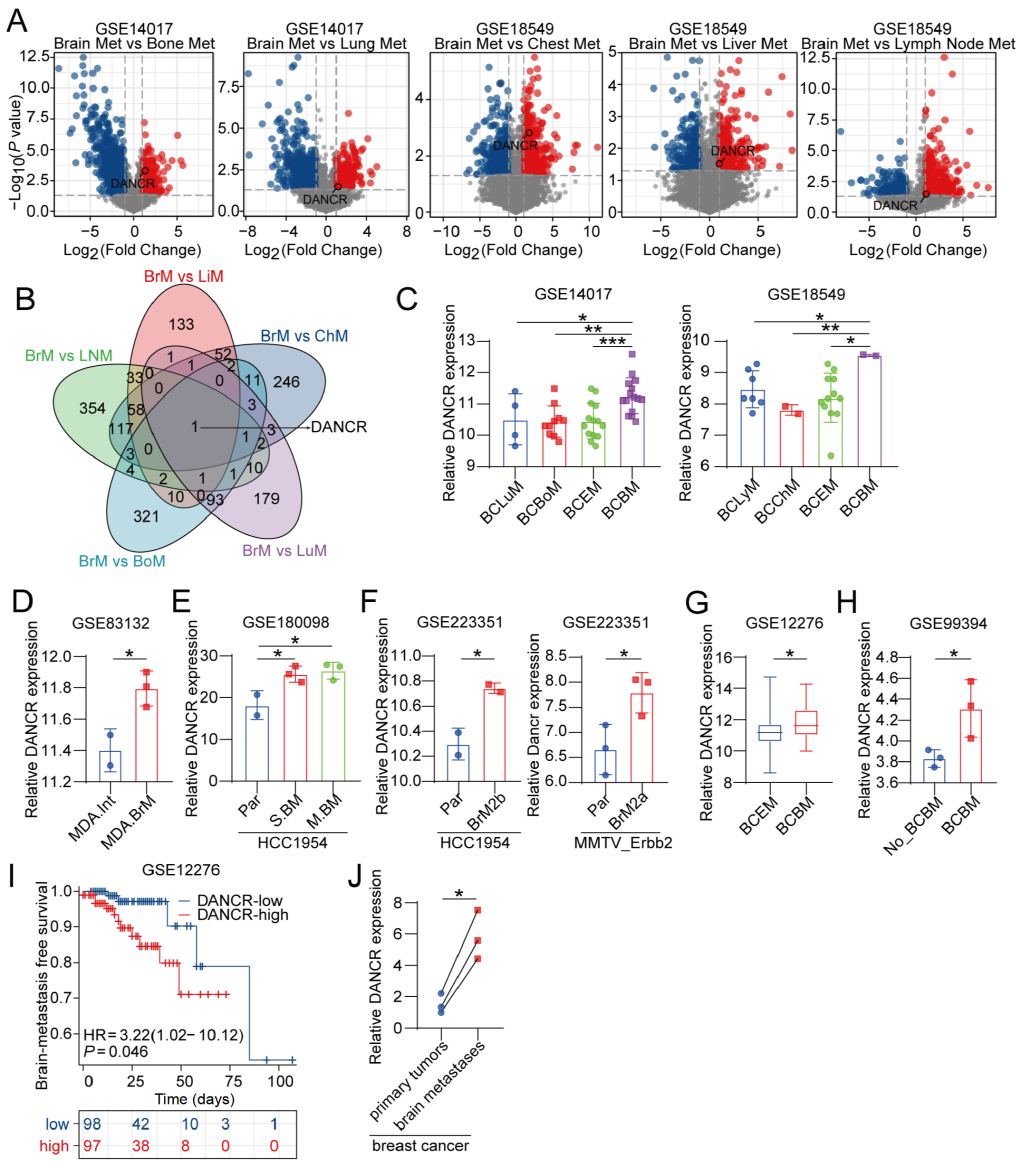
Figure 1 DANCR is associated with brain-specific metastasis in breast cancer (A) Differential gene expression analysis comparing brain metastases and multiple extracranial metastatic sites. (B) Venn diagram illustrating the overlap of upregulated genes across different metastatic cohorts. (C) DANCR expression levels in BCBM and extracranial metastases; n = 4–15. (D–F) DANCR expression levels in breast cancer brain metastatic sublines and their corresponding parental cells; n = 2–3. (G) Comparison of DANCR expression in primary tumors from breast cancer patients with and without brain metastases; n = 15–168. (H) Comparison of DANCR expression in blood samples from breast cancer patients with and without brain metastases; n = 3. (I) Kaplan-Meier analysis of brain metastasis-free survival stratified by DANCR expression in primary tumors. (J) DANCR expression levels in brain metastasis tissues and matched breast cancer tissues; n = 3. Data are presented as the mean ± SD. *P < 0.05, **P < 0.01, ***P < 0.001, determined by unpaired two-tailed Student’s t test or one-way ANOVA for multiple group comparisons.

Comparative transcriptomic analysis of the GSE14017 and GSE18549 datasets revealed differential gene expression profiles between BCBM and extracranial metastases involving the lung, liver, bone, chest wall, and lymph nodes ( Figure 1A). Venn diagram-based intersection analysis revealed that the long non-coding RNA DANCR was the only transcript consistently upregulated across cohorts ( Figure 1B). The expression of DANCR in the GSE14017 and GSE18549 datasets again revealed that DANCR was upregulated in BCBM compared with various extracranial metastases of breast cancer ( *P*  < 0.05, Figure 1C). Validation across GSE83132, GSE180098, and GSE223351 demonstrated consistent DANCR overexpression in both murine ( *P*  < 0.05) and human ( *P*  < 0.05) brain-tropic sublines compared with parental cells ( Figure 1D–F). Clinical correlation analyses of the GSE12276 and GSE99394 datasets revealed elevated DANCR levels in primary tumors ( *P*  < 0.05) and circulating tumor cells ( *P*  < 0.05) from BM-positive patients compared with those from BM-negative patients ( Figure 1G,H). Kaplan-Meier analysis of the GSE12276 data revealed reduced BM-free survival in patients with high DANCR expression (hazard ratio = 3.22, 95% confidence interval: 1.02–10.12, *P* = 0.046) ( Figure 1I). By using qRT-PCR, the expression of *DANCR* in three samples of brain metastasis tissues was determined to be upregulated compared with that in matched breast cancer tissues ( Figure 1J).

Collectively, these data demonstrated that DANCR was markedly upregulated in brain metastases of patients with breast cancer and that its elevated expression in patients with breast cancer specifically correlated with the prognostic risk of brain metastases.

### DANCR promotes breast cancer cell proliferation, migration and invasion *in vitro* and brain metastasis *in vivo*

**Figure 2:**
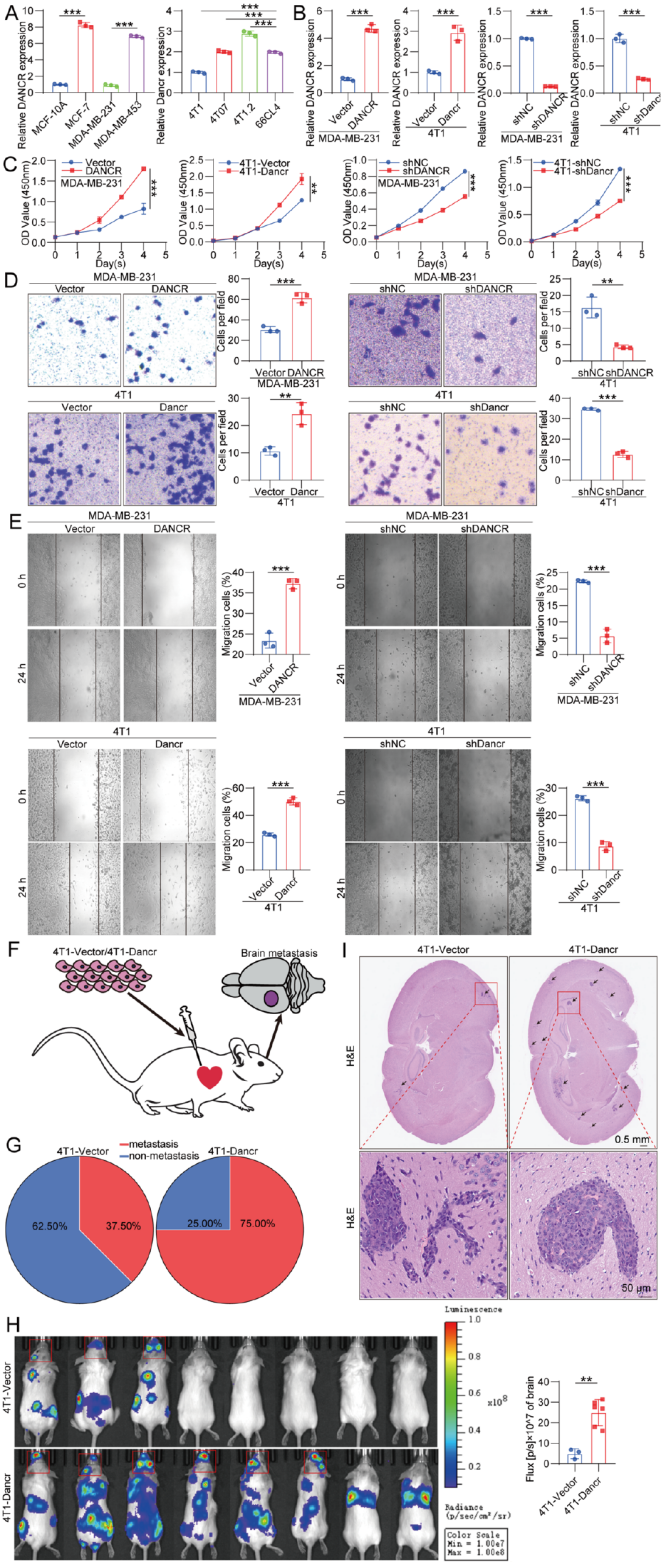
Figure 2 DANCR promotes breast cancer cell proliferation, migration and invasion *in vitro* and brain metastasis *in vivo* (A) Basal DANCR expression levels across different breast cancer cell lines; n = 3. (B) DANCR expression following overexpression and knockdown in MDA-MB-231 and 4T1 cells; n = 3. (C–E) Proliferation, migration, and invasion capacities were assessed in MDA-MB-231 and 4T1 cells following changes in DANCR via CCK-8, wound healing, and transwell assays, respectively; n = 3. (F) Diagram of the construction of the BCBM mouse model. (G) Incidence of brain metastases in the 4T1-Vector and 4T1-Dancr groups; n = 8. (H) Bioluminescence imaging analysis of mice injected with 4T1-Vector or 4T1-Dancr cells; n = 8. (I) Representative H&E staining image showing the brain metastatic tumor burden in mice injected with 4T1-Vector or 4T1-Dancr cells; n = 8. Data are presented as the mean ± SD. **P < 0.01, ***P < 0.001, determined by unpaired two-tailed Student’s t test or one-way ANOVA for multiple group comparisons.

qRT-PCR analysis revealed differences in basal DANCR expression across human and murine breast cancer cell lines. DANCR was notably more highly expressed in MCF-7 and MDA-MB-453 cells than in MCF-10A and MDA-MB-231 cells. Among murine cell lines, DANCR expression was the highest in 4T1.2 cells and the lowest in 4T1 cells ( Figure 2A). Consequently, for subsequent functional experiments, we selected MDA-MB-231 and 4T1 cells for overexpression and knockdown ( Figure 2B).

To investigate the role of DANCR in breast cancer progression, we conducted a series of functional assays. DANCR significantly enhanced the proliferation of breast cancer cells in the CCK-8 assay ( *P*  < 0.01, Figure 2C). Additionally, wound healing and transwell assays demonstrated that DANCR markedly increased the migration and invasion of breast cancer cells ( *P*  < 0.01, Figure 2D,E). According to western blot analysis, DANCR overexpression significantly upregulated PCNA and N-cadherin, whereas *DANCR* silencing upregulated E-cadherin ( Supplementary Figure S1).

For *in vivo* studies, a BCBM model was established in female BALB/c mice ( Figure 2F). The overexpression of DANCR significantly increased the percentage of BCBM, from 37.5% to 75%, as demonstrated by bioluminescence imaging ( Figure 2G,H). Bioluminescence imaging also revealed that DANCR notably increased both the brain and extracranial metastatic disease burdens ( Figure 2H). Interestingly, in the DANCR high-expression group, the photon counts in the brain metastases were significantly higher than those in the extracranial metastases. Conversely, in the DANCR low-expression group, the photon counts in the brain metastases were markedly lower than those in the extracranial metastases. H&E staining of brain sections revealed that upregulated DANCR caused a higher metastatic disease burden in the brain ( Figure 2I).

These findings suggest that DANCR plays a crucial role in the initiation and progression of BCBM.

### DANCR promotes BCBM by upregulating PTGS2

**Figure 3:**
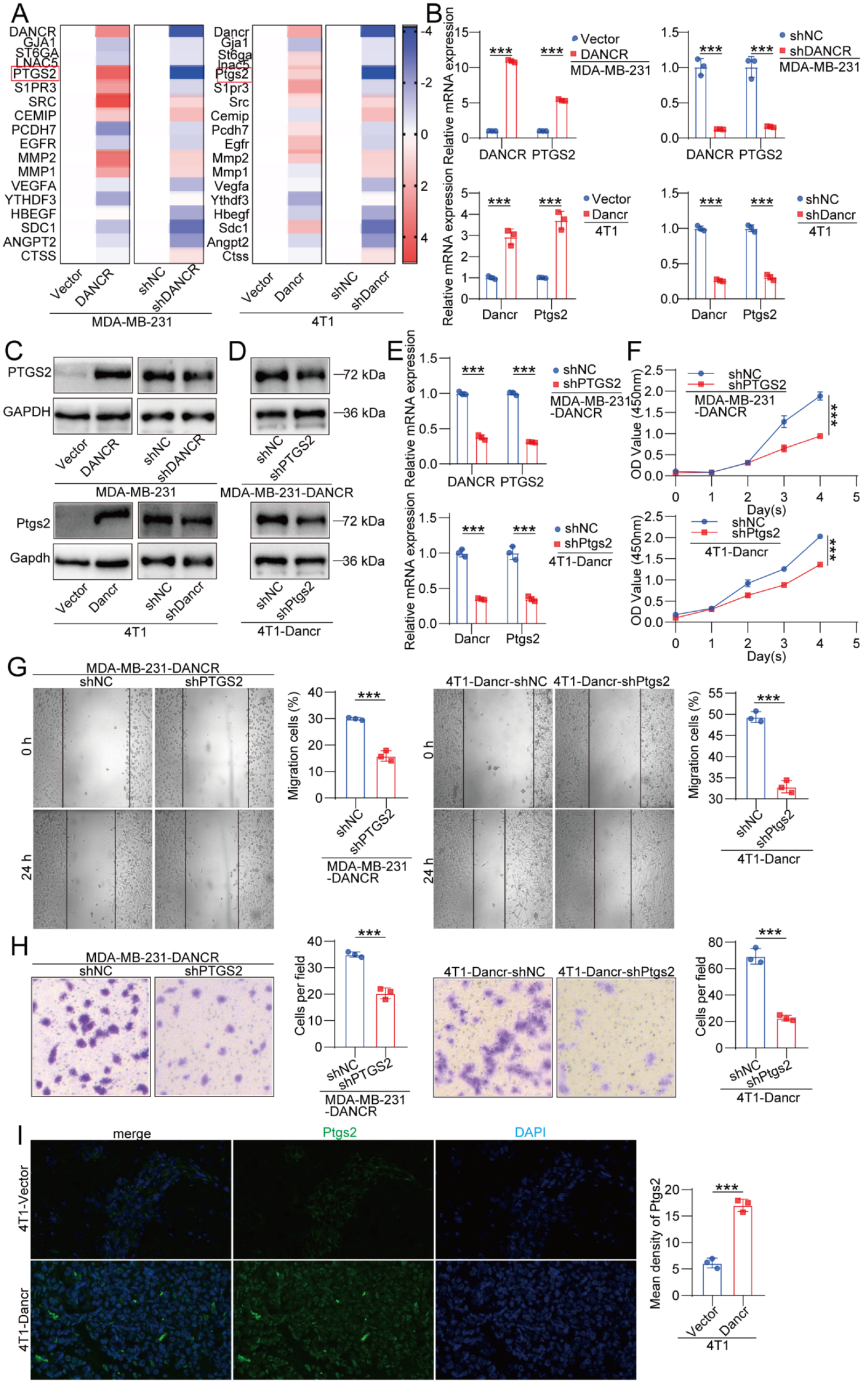
Figure 3 DANCR promotes BCBM through the upregulation of PTGS2 (A) Heatmap showing the expression levels of brain metastasis-associated genes; n = 3. (B,C) PTGS2 protein and mRNA and DANCR expression levels following DANCR modulation in MDA-MB-231 and 4T1 cells; n = 3. (D,E) PTGS2 protein and mRNA and DANCR expression levels following PTGS2 knockdown in DANCR-overexpressing cells; n = 3. (F–H) CCK-8, wound healing, and transwell assays showing the effects of PTGS2 silencing on DANCR-induced proliferation, migration, and invasion in MDA-MB-231 and 4T1 cells; n = 3. (I) Immunofluorescence staining showing PTGS2 expression in brain metastatic lesions; n = 3. Data are presented as the mean ± SD. ***P < 0.001, determined by unpaired two-tailed Student’s t test or one-way ANOVA for multiple group comparisons.

To elucidate potential downstream targets of DANCR in BCBM, we conducted qRT-PCR analysis of multiple genes implicated in metastatic progression. Among the candidates examined, PTGS2 expression was strongly positively correlated with DANCR levels in both MDA-MB-231 and 4T1 cells, suggesting that PTGS2 may mediate the functional effects of DANCR ( Figure 3A–C).

To further investigate this regulatory relationship, PTGS2 shRNA was introduced into stable DANCR-overexpressing cell lines, and the knockdown efficiency was confirmed by both qRT-PCR and western blot analysis ( Figure 3D,E). We subsequently evaluated the impact of PTGS2 silencing on breast cancer cell function using a series of functional assays. Notably, *PTGS2* knockdown significantly attenuated DANCR-induced increases in cell proliferation, migration, and invasion ( Figure 3F–H). Immunofluorescence staining of brain metastases further revealed that high DANCR expression was associated with elevated PTGS2 levels within metastatic lesions, supporting the regulatory link between DANCR and PTGS2 in the context of BCBM ( Figure 3I).

These data indicate that DANCR promotes BCBM via the upregulation of PTGS2.

### DANCR promotes PTGS2 expression by decoying miR-758-3p

**Figure 4:**
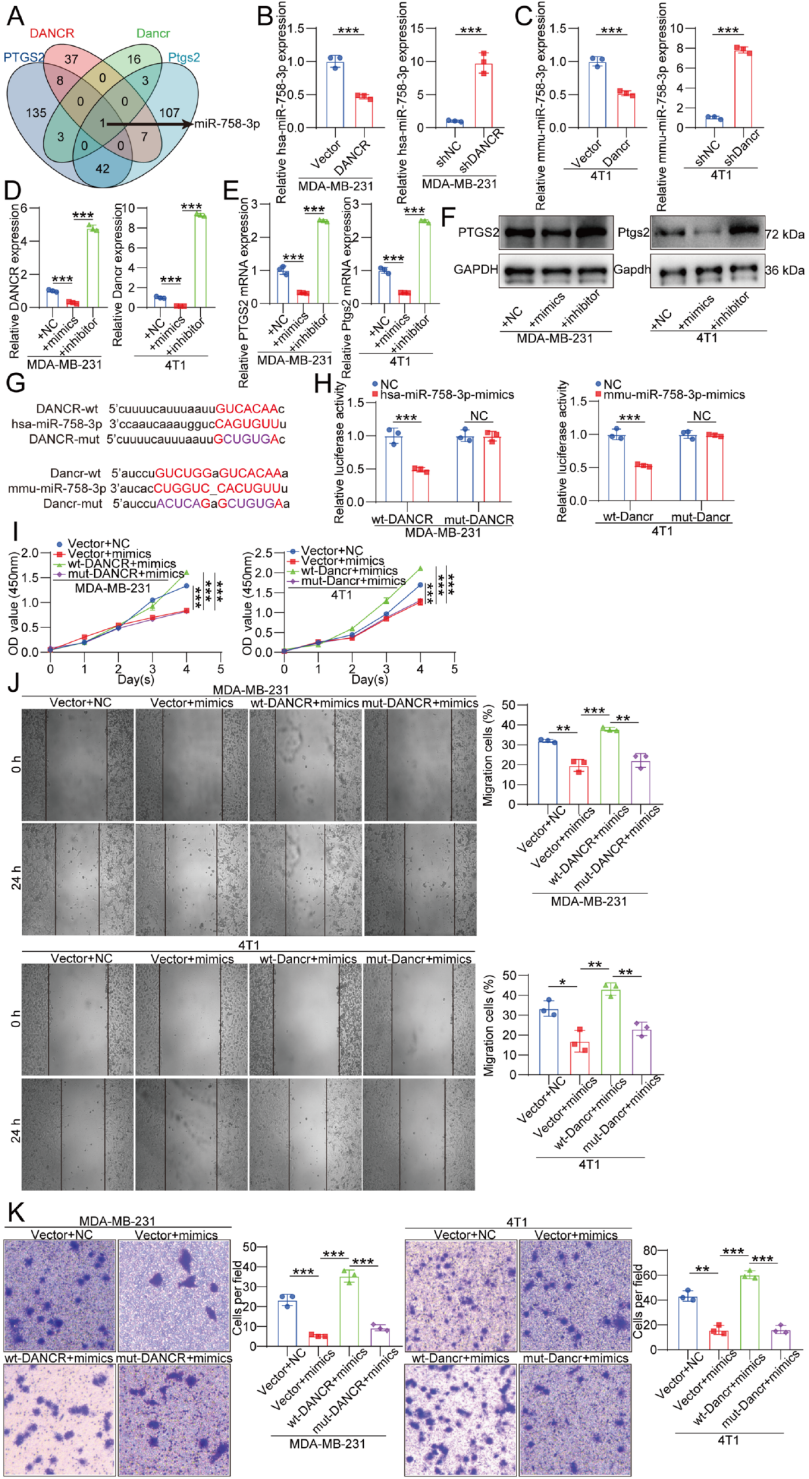
Figure 4 DANCR promotes PTGS2 expression by acting as a decoy for miR-758-3p (A) Venn diagram showing that both human and murine DANCR and PTGS2 share predicted binding sites for miR-758-3p. (B,C) Expression levels of miR-758-3p following DANCR modulation in MDA-MB-231 and 4T1 cells; n = 3. (D–F) Expression levels of DANCR and PTGS2 following miR-758-3p modulation in MDA-MB-231 and 4T1 cells; n = 3. (G) Schematic showing wild-type and mutated miR-758-3p binding sites in human and murine DANCR; n = 3. (H) Dual-luciferase reporter assay demonstrating reduced luciferase activity upon the binding of miR-758-3p to wild-type DANCR and the loss of this effect with mutated binding sites; n = 3. (I–K) CCK-8, wound healing, and Transwell assays demonstrating the effects of miR-758-3p on DANCR-induced proliferation, migration, and invasion in MDA-MB-231 and 4T1 cells; n = 3. Data are presented as the mean ± SD. *P < 0.05, **P < 0.01, ***P < 0.001, determined by unpaired two-tailed Student’s t test or one-way ANOVA for multiple group comparisons.

ENCORI database analysis revealed that both human and murine DANCR and PTGS2 contain conserved miRNA response elements for miR-758-3p ( Figure 4A) [33]. qRT-PCR and western blot analysis confirmed that DANCR negatively regulates miR-758-3p expression, whereas miR-758-3p reciprocally downregulates DANCR and PTGS2 levels ( Figure 4B–F).

A dual-luciferase reporter assay validated the direct interaction between DANCR and miR-758-3p. Co-transfection of wild-type (wt) DANCR with miR-758-3p mimics resulted in a significant reduction in fluorescence intensity compared with that of the NC mimics. In contrast, mutation of the predicted miR-758-3p binding site on DANCR abolished this difference, confirming the specificity of their interaction ( Figure 4G,H).

Further functional assays, including CCK-8, wound healing, and Transwell assays, demonstrated that wt-DANCR effectively reversed the inhibitory effects of miR-758-3p on cell proliferation, migration, and invasion. However, mutation of the miR-758-3p binding site on DANCR abrogated this rescue effect, reinforcing the role of DANCR ( Figure 4I–K).

**Figure 5:**
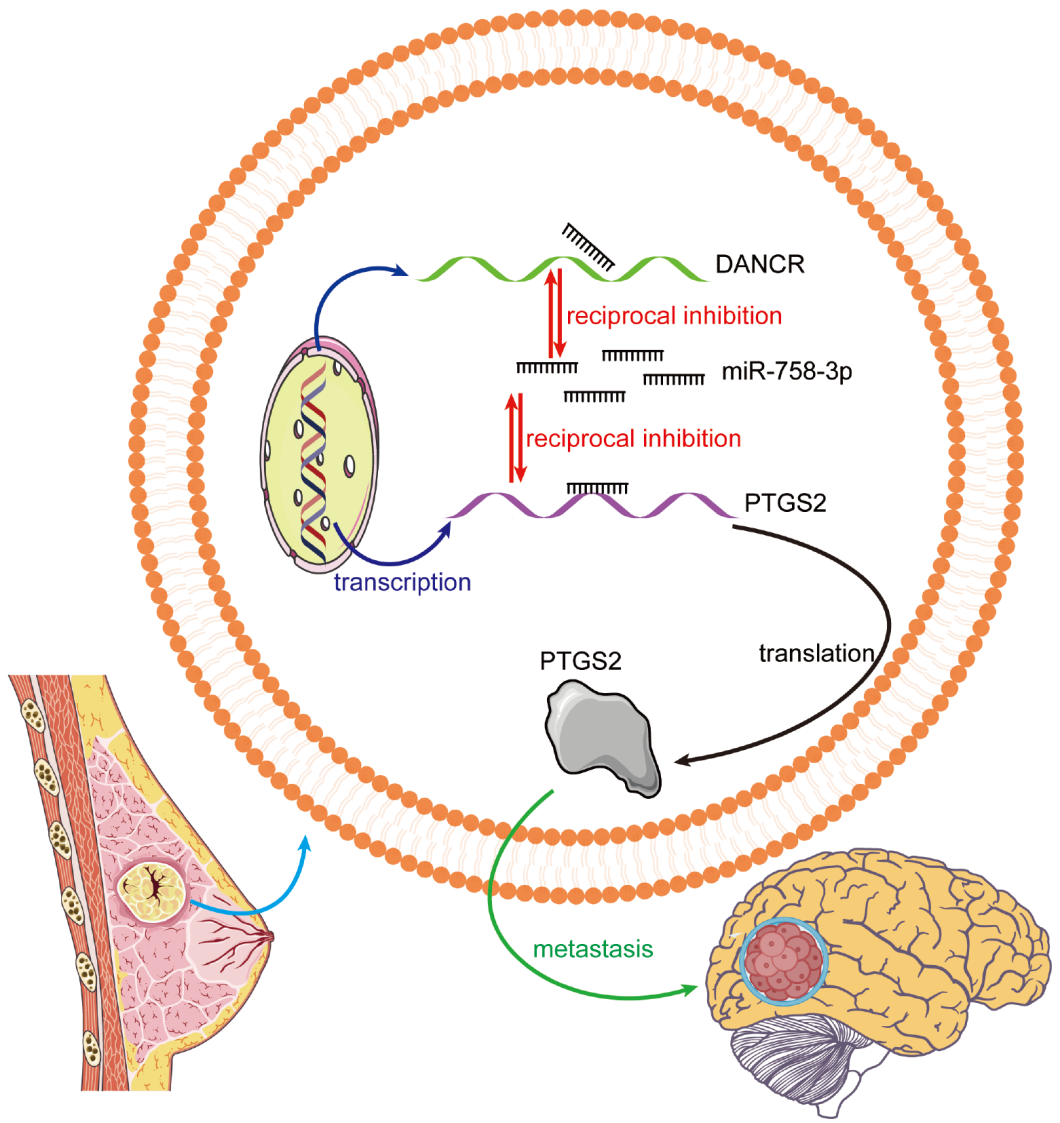
Figure 5 DANCR promotes PTGS2-mediated brain metastasis in breast cancer by decoying miR-758-3p Upregulated DANCR competitively binds to more miR-758-3p, reducing the amount of miR-758-3p available for binding to PTGS2 mRNA, thereby resulting in increased PTGS2 expression. By enhancing the ability of tumor cells to cross the BBB, elevated PTGS2 promotes greater infiltration of tumor cells into the brain.

Taken together, these results indicate that DANCR acts as a competing endogenous RNA (ceRNA) that regulates PTGS2 expression via miR-758-3p sequestration ( Figure 5).

## Discussion

With advances in systemic therapies and prolonged survival of breast cancer patients, the increasing incidence of BCBM remains a significant clinical challenge. The identification of additional therapeutic targets is urgently needed. Bioinformatics analysis revealed that DANCR was significantly upregulated in BCBM and was specifically correlated with the prognostic risk of BCBM. *In vitro* and *in vivo* experiments confirmed that DANCR promotes the occurrence and progression of BCBM. PTGS2 expression consistently varied in parallel with DANCR levels, and silencing PTGS2 reversed the protumor effects induced by DANCR *in vitro*. Furthermore, the interaction between DANCR and PTGS2 is mediated by miR-758-3p.

DANCR is an oncogenic lncRNA. Previous studies have shown that DANCR plays an oncogenic role in various malignancies, including nasopharyngeal carcinoma, lung cancer, colorectal cancer, liver cancer, breast cancer, and prostate cancer [ 34, 35] . High DANCR levels are correlated with poorer overall survival, larger tumor size, and more advanced disease stage [ 36– 40] . Our results were in line with these previously reported findings. Numerous studies have indicated that DANCR is closely linked to biological processes such as abnormal proliferation, invasion, metastasis, angiogenesis, energy metabolism reprogramming, and inflammatory phenotypes [35]. In our study, we observed significant upregulation of DANCR expression in BCBM compared with that in various extracranial metastases. *In vitro* and *in vivo* experiments confirmed that DANCR increases the incidence and burden of breast cancer metastases, particularly brain metastases. These findings suggest that DANCR not only enhances the proliferative and metastatic potential of breast cancer cells but also increases their tropism toward the brain.

PTGS2 is one of two genes encoding enzymes that catalyze the conversion of arachidonate to prostaglandin. Bos *et al*. [19] demonstrated that PTGS2 facilitates breast cancer cell extravasation through the BBB. Wu *et al*. [41] reported that PTGS2 in brain metastatic cells induces prostaglandins to upregulate MMP1 and activates astrocytes to express CCL7, promoting brain metastasis and BBB dysfunction. In our study, we identified a positive correlation between DANCR and PTGS2 expression both *in vitro* and *in vivo*. Silencing PTGS2 effectively reversed the DANCR-induced increase in breast cancer cell proliferation, migration, and invasion. These findings suggest that DANCR promotes BCBM by upregulating PTGS2.

In the ceRNA mechanism, a classical regulatory process involving non-coding RNAs, lncRNAs act as molecular sponges for miRNAs. Specifically, lncRNAs competitively bind to miRNAs, thereby reducing the amount of miRNA available to bind to target mRNAs and alleviating their repression of target mRNAs. Harati *et al*. reported that miR-26b-5p and miR-101-3p synergistically inhibit PTGS2/MMP-1 signalling and suppress the transendothelial migration of breast cancer cells by targeting PTGS2 [ 42, 43] . Wang *et al*. [44] demonstrated that DANCR regulates the apoptosis and autophagy of breast cancer cells via the DANCR-miR-758-3p-PAX6 network. Zhang *et al*. [45] reported that DANCR performs oncogenic functions in non-small cell lung cancer by regulating miR-758-3p. In our study, the ENCORI database predicted that miR-758-3p could target both DANCR and PTGS2 in human and murine tumor cells. Overexpression of DANCR alleviated the inhibitory effects of miR-758-3p on cell proliferation, migration, and invasion, whereas mutation of the predicted binding site on DANCR abrogated this effect. On the basis of these results, we hypothesize that upregulated DANCR competitively binds to more miR-758-3p, reducing the amount of miR-758-3p available for binding to PTGS2 mRNA, thereby resulting in increased PTGS2 expression. By enhancing the ability of tumor cells to cross the BBB, elevated PTGS2 promotes greater infiltration of tumor cells into the brain.

In conclusion, our study revealed a novel regulatory axis in which DANCR functions as a ceRNA to sequester miR-758-3p, thereby upregulating PTGS2 expression and promoting BCBM. These findings not only enhance our mechanistic understanding of BCBM but also provide potential therapeutic targets for the development of novel strategies aimed at preventing and treating this devastating complication.

## Supporting information

25181Supplementary_Data(1)

25181Figure_S1

## Supplementary Data

Supplementary data is available at *Acta Biochimica et Biophysica Sinica* online.
