# Supplementary material for: The lncRNA DANCR promotes breast cancer brain metastasis by acting as a ceRNA for miR-758-3p to regulate PTGS2 expression: DANCR/miR-758-3p/PTGS2 axis drives breast cancer brain metastasis
Source: Acta Biochim Biophys Sin (Shanghai). 2025 May 15;58(3):610–20. doi: 10.3724/abbs.2025082 (PMC13059747; doi:10.3724/abbs.2025082)
Supplement: 25181Supplementary_Data(1) [file 25181Supplementary_Data(1).docx]

| **Supplementary Table S1. Sequences of primers for RT-qPCR and shRNA** | | |
| --- | --- | --- |
| Gene |  | Sequence (5′→3′) |
| shDANCR | F: | CCGGAGGAGCTAGAGCAGTGACAATCTCGAGATTGTCACTGCTCTAGCTCCTTTTTTT |
|  | R: | AATTAAAAAAGGAGCTAGAGCAGTGACAATCTCGAGATTGTCACTGCTCTAGCTCCT |
| shNC | F: | CCGGTCCTAAGGTTAAGTCGCCCTCGCTCGAGCGAGGGCGACTTAACCTTAGGTTTTT |
|  | R: | AATTAAAAATCCTAAGGTTAAGTCGCCCTCGCTCGAGCGAGGGCGACTTAACCTTAGG |
| shDancr | F: | CCGGGCATGTAGTGACCAGGTTTGCCTCGAGGCAAACCTGGTCACTACATGCTTTTT |
|  | R: | AATTAAAAAGCATGTAGTGACCAGGTTTGCCTCGAGGCAAACCTGGTCACTACATGC |
| shPTGS2 | F: | CCGGGCTGAATTTAACACCCTCTATCTCGAGATAGAGGGTGTTAAATTCAGCTTTTTT |
|  | R: | AATTAAAAAGCTGAATTTAACACCCTCTATCTCGAGATAGAGGGTGTTAAATTCAGC |
| shPtgs2 | F: | CCGGCCGTACACATCATTTGAAGAACTCGAGTTCTTCAAATGATGTGTACGGTTTTTT |
|  | R: | AATTAAAAACCGTACACATCATTTGAAGAACTCGAGTTCTTCAAATGATGTGTACGG |
| DANCR | F: | GCGCCACTATGTAGCGGGTT |
|  | R: | TCAATGGCTTGTGCCTGTAGTT |
| Dancr | F: | CTTGGTGGTGAGTGTCCTCG |
|  | R: | AGAACTGCTCTACCCCTCCG |
| PTGS2 | F: | CCAAAATCGTATTGCTGCTG |
|  | R: | CTGGGTAATTCCATGTTCC |
| Ptgs2 | F: | TTCAACACACTCTATCACTGGC |
|  | R: | AGAAGCGTTTGCGGTACTCAT |
| GAPDH | F: | CTGGGCTACACTGAGCACC |
|  | R: | AAGTGGTCGTTGAGGGCAATG |
| Gapdh | F: | AGGTCGGTGTGAACGGATTTG |
|  | R: | TGTAGACCATGTAGTTGAGGTCA |
| hsa-miR-758-3p | F: | TTTGTGACCTGGTCCACTAACC |
|  | R: | CAGTGCGTGTCGTGGAGT |
| U6 | F: | CTCGCTTCGGCAGCACA |
|  | R: | AACGCTTCACGAATTTGCGT |
| mmu-miR-758-3p | F: | GTACTTTGTGACCTGGTCCACTA |
|  | R: | CAGTGCGTGTCGTGGAGT |
| PCDH7 | F: | TGATCTTCGACGAGAACGAGT |
|  | R: | CGTTGATGTCAAGCACGATGA |
| GJA1 | F: | GGTGACTGGAGCGCCTTAG |
|  | R: | GCGCACATGAGAGATTGGGA |
| ST6GALNAC5 | F: | CACTGGCTGGTTTACAATGACA |
|  | R: | GTCCTCGCTCATGGGAGAG |
| SDC1 | F: | CTGCCGCAAATTGTGGCTAC |
|  | R: | TGAGCCGGAGAAGTTGTCAGA |
| CRYAB | F: | AGGTGTTGGGAGATGTGATTGA |
|  | R: | GGATGAAGTAATGGTGAGAGGGT |
| EGFR | F: | TTGCCGCAAAGTGTGTAACG |
|  | R: | GTCACCCCTAAATGCCACCG |
| VEGFA | F: | AGGGCAGAATCATCACGAAGT |
|  | R: | AGGGTCTCGATTGGATGGCA |
| HBEGF | F: | ATCGTGGGGCTTCTCATGTTT |
|  | R: | TTAGTCATGCCCAACTTCACTTT |
| CEMIP | F: | GAACCCGGCACATCCTGATT |
|  | R: | GATCCGGCTGAATACCTTCATC |
| CTSS | F: | TGTAGATGCGCGTCATCCTTC |
|  | R: | CCAACCACAAGTACACCATGAT |
| ANGPT2 | F: | AACTTTCGGAAGAGCATGGAC |
|  | R: | CGAGTCATCGTATTCGAGCGG |
| MMP1 | F: | GGGGCTTTGATGTACCCTAGC |
|  | R: | TGTCACACGCTTTTGGGGTTT |
| SRC | F: | GTGTCTTCTCTCTCTCCTGCCA |
|  | R: | GAGTTGAAGCCTCCGAACAG |
| MMP2 | F: | TACAGGATCATTGGCTACACACC |
|  | R: | GGTCACATCGCTCCAGACT |
| S1PR3 | F: | GTGATCCTCTACGCACGCATC |
|  | R: | CGCTCCGAGTTGTTGTGGT |
| Pcdh7 | F: | TCAGCCTTGAGTCTGGTTCTG |
|  | R: | CATTCGTTCTCGTCGAAGATCAT |
| Gja1 | F: | CTGAGTGCGGTCTACACCTG |
|  | R: | GAGCGAGAGACACCAAGGAC |
| St6galnac5 | F: | AGACAGAGTGTGTTATCCGCA |
|  | R: | TGTAGGTTGTTGTACGCCTGG |
| Sdc1 | F: | AACGGGCCTCAACAGTCAG |
|  | R: | CCGTGCGGATGAGATGTGA |
| Cryab | F: | GTTCTTCGGAGAGCACCTGTT |
|  | R: | GAGAGTCCGGTGTCAATCCAG |
| Egfr | F: | ATGAAAACACCTATGCCTTAGCC |
|  | R: | TAAGTTCCGCATGGGCAGTTC |
| Vegfa | F: | GCACATAGAGAGAATGAGCTTCC |
|  | R: | CTCCGCTCTGAACAAGGCT |
| Hbegf | F: | CGGGGAGTGCAGATACCTG |
|  | R: | TTCTCCACTGGTAGAGTCAGC |
| Cemip | F: | TGGCAGGAGCCACTACTACTG |
|  | R: | CCCTTTCGTTCTGGGCTTTTAAT |
| Ctss | F: | CCATTGGGATCTCTGGAAGAAAA |
|  | R: | TCATGCCCACTTGGTAGGTAT |
| Angpt2 | F: | CCTCGACTACGACGACTCAGT |
|  | R: | TCTGCACCACATTCTGTTGGA |
| Mmp13 | F: | CTTCTTCTTGTTGAGCTGGACTC |
|  | R: | CTGTGGAGGTCACTGTAGACT |
| Src | F: | GAACCCGAGAGGGACCTTC |
|  | R: | GAGGCAGTAGGCACCTTTTGT |
| Mmp2 | F: | CAAGTTCCCCGGCGATGTC |
|  | R: | TTCTGGTCAAGGTCACCTGTC |
| S1pr3 | F: | ACTCTCCGGGAACATTACGAT |
|  | R: | CAAGACGATGAAGCTACAGGTG |

**
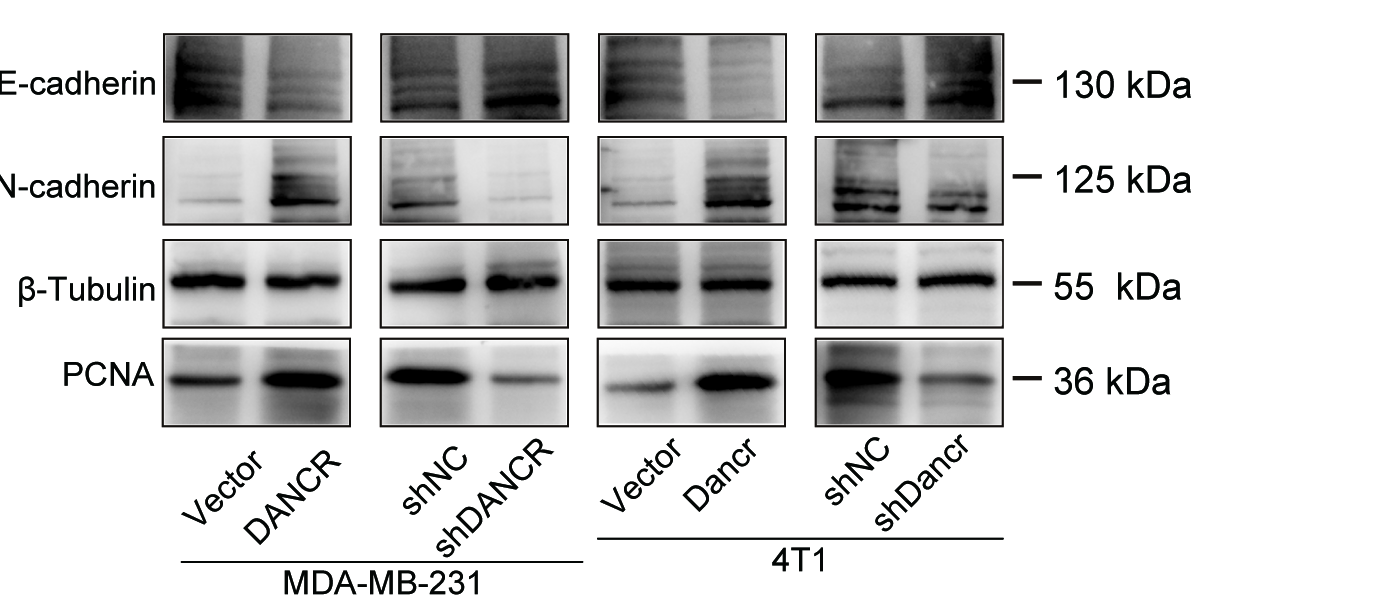
**

**Supplementary Figure S1. Changes of key proliferative and metastatic markers of breast cancer cells**
