# Supplementary figures and images for: The lncRNA DANCR promotes breast cancer brain metastasis by acting as a ceRNA for miR-758-3p to regulate PTGS2 expression: DANCR/miR-758-3p/PTGS2 axis drives breast cancer brain metastasis
Source: Acta Biochim Biophys Sin (Shanghai). 2025 May 15;58(3):610–20. doi: 10.3724/abbs.2025082 (PMC13059747; doi:10.3724/abbs.2025082)

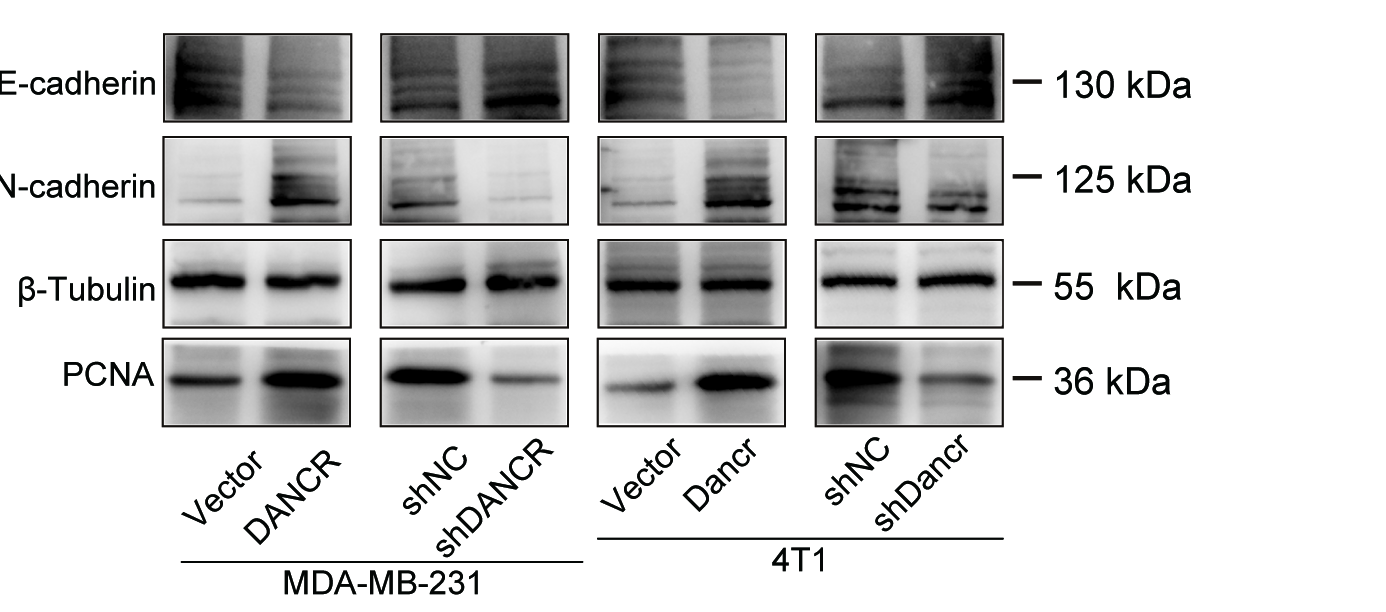

Supplement: 25181Figure_S1 [file 25181Figure_S1.tif]
